# Supplementary material for: Synthetic data in cancer and cerebrovascular disease research: A novel approach to big data
Source: PLoS One. 2024 Feb 7;19(2):e0295921. doi: 10.1371/journal.pone.0295921 (PMC10849264; doi:10.1371/journal.pone.0295921)
Supplement: S1 File — (PDF) [file pone.0295921.s001.pdf]

Supplemental Information

Number of Table: 4

S1 Table. Search terms utilized for identifying cancer subtypes for Cohort 1: Cancer and Ischemic Stroke cohort

- Carcinoma in situ of breast
- Carcinoma in situ of cervix uteri
- Carcinoma in situ of middle ear and respiratory system
- Carcinoma in situ of oral cavity, oesophagus and stomach
- Carcinoma in situ of other and unspecified digestive organs
- Carcinoma in situ of other and unspecified genital organs
- Carcinoma in situ of other and unspecified sites
- Malignant immunoproliferative diseases
- Malignant neoplasm of accessory sinuses
- Malignant neoplasm of adrenal gland
- Malignant neoplasm of anus and anal canal
- Malignant neoplasm of base of tongue
- Malignant neoplasm of bladder
- Malignant neoplasm of bone and articular cartilage of limbs
- Malignant neoplasm of bone and articular cartilage of other and unspecified sites
- Malignant neoplasm of breast
- Malignant neoplasm of bronchus and lung
- Malignant neoplasm of cervix uteri
- Malignant neoplasm of colon
- Malignant neoplasm of corpus uteri
- Malignant neoplasm of eye and adnexa
- Malignant neoplasm of floor of mouth
- Malignant neoplasm of gallbladder
- Malignant neoplasm of gum
- Malignant neoplasm of heart, mediastinum and pleura
- Malignant neoplasm of hypopharynx
- Malignant neoplasm of kidney, except renal pelvis
- Malignant neoplasm of larynx
- Malignant neoplasm of lip
- Malignant neoplasm of liver and intrahepatic bile ducts
- Malignant neoplasm of nasal cavity and middle ear
- Malignant neoplasm of nasopharynx
- Malignant neoplasm of oesophagus
- Malignant neoplasm of oropharynx
- Malignant neoplasm of other and ill-defined digestive organs
- Malignant neoplasm of other and ill-defined sites
- Malignant neoplasm of other and ill-defined sites in the lip, oral cavity and pharynx
- Malignant neoplasm of other and ill-defined sites in the respiratory system and intrathoracic organs

- Malignant neoplasm of other and unspecified female genital organs
- Malignant neoplasm of other and unspecified major salivary glands
- Malignant neoplasm of other and unspecified male genital organs
- Malignant neoplasm of other and unspecified parts of biliary tract
- Malignant neoplasm of other and unspecified parts of mouth
- Malignant neoplasm of other and unspecified parts of tongue
- Malignant neoplasm of other and unspecified urinary organs
- Malignant neoplasm of other connective and soft tissue
- Malignant neoplasm of other endocrine glands and related structures
- Malignant neoplasm of ovary
- Malignant neoplasm of palate
- Malignant neoplasm of pancreas
- Malignant neoplasm of parotid gland
- Malignant neoplasm of penis
- Malignant neoplasm of peripheral nerves and autonomic nervous system
- Malignant neoplasm of placenta
- Malignant neoplasm of prostate
- Malignant neoplasm of pyriform sinus
- Malignant neoplasm of rectosigmoid junction
- Malignant neoplasm of rectum
- Malignant neoplasm of renal pelvis
- Malignant neoplasm of retroperitoneum and peritoneum
- Malignant neoplasm of small intestine
- Malignant neoplasm of stomach
- Malignant neoplasm of testis
- Malignant neoplasm of thymus
- Malignant neoplasm of thyroid gland
- Malignant neoplasm of tonsil
- Malignant neoplasm of trachea
- Malignant neoplasm of ureter
- Malignant neoplasm of uterus, part unspecified
- Malignant neoplasm of vagina
- Malignant neoplasm of vulva
- Malignant neoplasm without specification of site
- Malignant neoplasms of independent (primary) multiple sites
- Multiple myeloma and malignant plasma cell neoplasms
- Other and unspecified malignant neoplasms of lymphoid, haematopoietic and related tissue
- Secondary and unspecified malignant neoplasm of lymph nodes
- Secondary malignant neoplasm of other and unspecified sites
- Secondary malignant neoplasm of respiratory and digestive organs

S2 Table. Distribution of the types of cancer in synthetic cohort #1: ischemic stroke patients with cancer

Cancer Type	Frequency	Percentage
Lung	242	18.98%
Secondary malignancies	183	14.35%
Colorectal	152	11.92%
Bladder	95	7.45%
Immunoproliferative disease	87	6.82%
Censored	80	6.27%
Unspecified	79	6.20%
Prostate	78	6.12%
Esophageal + upper GI	56	4.39%
Breast	49	3.84%
Pancreatic	43	3.37%
Urogynecologic	38	2.98%
Kidney	30	2.35%
Head and neck cancer	22	1.73%
Bone and cartilage	18	1.41%
Hepatobiliary	14	1.10%
Melanoma	9	0.71%

S3 Table. List of codes used to select patients with ischemic stroke using the International Classification of Diseases (ICD) 10th revision.

	**ICD-10 coding**
**Ischemic Stroke**	I63.00, I63.01x, I63.02, I63.03x, I63.09, I63.10, I63.11x, I63.12, I63.13x,<br>I63.19, I63.20, I63.21x, I63.22, I63.23x, I63.29, I63.30, I63.31x, I63.32x,<br>I63.33x, I63.34x, I63.39, I63.40, I63.41x, I63.42x, I63.43x, I63.44x, I63.49,<br>I63.50, I63.51x, I63.52x, I63.53x, I63.54x, I63.59, I63.6, I63.8, I63.9

S4 Table. Inflation-adjusted median costs per year for the cancer and stroke (A) and no cancer and stroke cohorts (B)

YEAR	ORIGINAL MEDIAN VALUES	INFLATION-ADJUSTED MEDIAN EQUIVALENT (2022)	ORIGINAL MEAN VALUES	INFLATION-ADJUSTED MEAN EQUIVALENT (2022)
2005	$7,505.24	$10,605.54	$7,505.24	$10,605.54
2006	$8,176.14	$11,331.19	$9,436.24	$13,077.53
2007	$5,874.77	$7,966.50	$9,587.93	$13,001.74
2008	$6,782.39	$8,964.14	$14,693.58	$19,420.19
2009	N/A	N/A	N/A	N/A
2010	$16,383.30	$21,263.13	$27,625.31	$35,853.62
2011	$4,750.74	$5,990.92	$6,107.72	$7,702.14
2012	$11,219.45	$13,939.04	$15,661.87	$19,458.30
2013	$10,212.74	$12,574.64	$17,707.16	$21,802.30
2014	$4,314.73	$5,210.76	$9,630.88	$11,630.91
2015	$19,823.37	$23,675.30	$18,557.10	$22,162.98
2016	$13,911.32	$16,381.55	$19,929.50	$23,468.39
2017	$11,185.63	$12,969.84	$25,279.86	$29,312.23
2018	$10,475.83	$11,873.65	$20,952.13	$23,747.84
2019	$9,946.43	$11,058.09	$11,728.51	$13,039.34
TOTAL			$15,314.50	$ 20,686.29

YEAR	ORIGINAL MEDIAN VALUES	INFLATION-ADJUSTED MEDIAN EQUIVALENT (2022)	ORIGINAL MEAN VALUES	INFLATION-ADJUSTED MEAN EQUIVALENT (2022)
2005	6068.76	8575.66	$15,701.71	$ 22,187.83
2006	5467.975	7577.98	$15,865.69	$ 21,988.01
2007	10218.88	13857.35	$18,059.32	$ 24,489.41
2008	9238.21	12242.04	$16,688.15	$ 22,114.36
2009	7389.82	9766.96	$12,792.01	$ 16,906.92

**2010**	10752.98	13955.80	$16,648.83	$ 21,607.75
**2011**	6746.39	8507.541	$11,254.43	$ 14,192.41
**2012**	8160.92	10139.12	$15,893.87	$ 19,746.54
**2013**	7719.48	9504.77	$11,372.40	$ 14,002.50
**2014**	8745.705	10561.91	$14,551.89	$ 17,573.85
**2015**	9061.67	10822.47	$14,370.74	$ 17,163.16
**2016**	8646.61	10181.99	$14,221.18	$ 16,746.44
**2017**	8278.39	9598.87	$15,960.06	$ 18,505.83
**2018**	6627.9	7512.28	$13,569.72	$ 15,380.37
**2019**	7099.4	7892.86	$9,211.71	$ 10,241.25
**TOTAL**			$14,410.78	$17,295.31
