# Supplementary material for: Synthetic data in cancer and cerebrovascular disease research: A novel approach to big data
Source: PLoS One. 2024 Feb 7;19(2):e0295921. doi: 10.1371/journal.pone.0295921 (PMC10849264; doi:10.1371/journal.pone.0295921)
Supplement: Attachment — Submitted filename: PLOS ONE Revisions Response to Reviewers V2.docx [file pone.0295921.s004.docx]

*PLOS ONE Revisions – Response to Reviewers*

*Review Comments to the Author

Please use the space provided to explain your answers to the questions above. You may also include additional comments for the author, including concerns about dual publication, research ethics, or publication ethics. (Please upload your review as an attachment if it exceeds 20,000 characters)*

*Reviewer #1: The authors have addressed the comments from the reviewer and may now be accepted. The authors are to be commended for this manuscrip.t*

Thank you for taking the time to review our manuscript.

*Reviewer #2: Thank you for the opportunity to review. This is truly novel and interesting manuscript.

Minor comments and questions to the authors:
1. CCI in outcome analyses: since you're investigating cancer patients and CCI contains cancer variable, have you adjusted CCI to remove cancer and re-calculate CCI?*

1. Thank you for this suggestion. The Charlson Comorbidity Index was automatically calculated by MDClone and therefore the cancer variable was not removed and re-calculated. This limitation has been added to the discussion section of our manuscript: “There were also some automatically calculated variables (i.e. the Charlson Comorbidity Index), where the individual components of the score could not be individually analyzed.”

*2. Looking at your original dataset time horizon (2002-2019), which is very long, have you considered adjusting costs for inflation in your analyses to make comparisons valid? Current cost estimates presented in the manuscript should be adjusted for inflation.?*

Thank you for this suggestion. We have broken down the median cost associated with encounters by year and adjusted each year’s median cost for inflation based on the 2022 Canadian consumer price index (Statistics Canada. Table 18-10-0005-01 Consumer Price Index, annual average, not seasonally adjusted). We have presented this data in the supplemental materials document S4 table. Pooling of the median values from each year was not undertaken as medians are not amenable to further mathematic calculations. However, we have also calculated the mean cost associated with encounters from each year and presented the original mean by year as well as the inflation-adjusted cost (adjusted for 2022), and presented the total mean sum for the cancer cohort (S4 Table A) and the non-cancer cohort (S4 Table B). The original mean for the cancer cohort was $15,314.50 and the inflation-adjusted cost was $20,686.29. Comparatively the original mean for the non-cancer cohort was $14,410.78 and after adjusting for inflation, the 2022 equivalent would be $17,295.31. Our conclusions are therefore similar – that the cancer cohort has higher costs associated with their encounter compared to the non-cancer cohort. We did not present these inflation-adjusted means in the main text of our manuscript because cost was not a normally distributed value and therefore presentation of the original median values would be more statistically correct. We have also added this to the text of our main manuscript: “We also calculated the median and average costs associated with stroke encounters for each year between 2005 – 2019 and adjusted the costs for inflation based on the 2022 Canadian consumer price index.^28^ The average costs associated with encounters in the cancer and stroke cohort was consistently higher compared to encounters in the non-cancer and stroke cohort (Supplemental Materials S4 Table).”

We hope this is satisfactory to the reviewer.

*3. How was stroke encounter defined, and how would the audience know whether the cost of an encounter can truly be associated with stroke? Also, how were indirect costs calculated*

Stroke encounters were identified using International Classification of Diseases 10^th^ edition (ICD-10) codes. The list of codes can be found now in Supplemental Materials Table S3. For Emergency Department visits, encounters were only included if stroke was the “most responsible diagnosis” associated with the visit. For hospitalizations, encounters were included if ischemic stroke was defined as the “primary problem”.

Information regarding costs were available for all inpatient encounters from April 2002, and for ED encounters, from April 2011. Direct costs were defined as all expenses in direct functional centers related to patient care, including salaries, supplies, and equipment amortization. Indirect costs included overhead allocation based on the percentage of the activity in the functional center, and consisted of costs not directly related to the patient, such as human resources, finance, health records, administration fees, building maintenance, etc.

The above statements have been added to our revised manuscript.
